# Bacillus Calmette-Guérin-related cold thigh abscess as an unusual cause of thigh swelling in infants following BCG vaccine administration: a case series

**DOI:** 10.1186/1752-1947-5-472

**Published:** 2011-09-22

**Authors:** Mohammad Al Namshan, Omar Oda, Jameela Almaary, Saud Al Jadaan, Stanley Crankson, Esam Al Banyan, Mohammad Al Shaalan, Mohammad Zamakhshary

**Affiliations:** 1 Division of Pediatric Surgery, Department of Surgery, King Abdulaziz Medical City, King Saud bin Abdulaziz University for Health Sciences, Riyadh, Saudi Arabia; 2 Division of Pediatric Infectious Diseases, Department of Pediatrics, King Abdulaziz Medical City, King Saud bin Abdulaziz University for Health Sciences, Riyadh, Saudi Arabia

## Abstract

**Introduction:**

Thigh swelling in an infant can be a symptom of a simple benign condition or a life-threatening condition. We observed a cluster of thigh swelling episodes in infants in which the cause was Bacillus Calmette-Guérin-related cold thigh abscess. We report this unusual case series to raise awareness about this diagnosis.

**Case presentations:**

We performed a retrospective review of five infants (four boys and one girl) who presented with Bacillus Calmette-Guérin-related left thigh abscess. The swelling was noticed by the parents at a mean period of three months prior to presentation. The ages at presentation were five, five, eight and nine months for the boys, and six months for the girl. All of the patients were healthy Saudi infants, and received the Bacillus Calmette-Guérin vaccine at birth. Clinically, all of the patients were well and did not demonstrate signs of systemic infection. All patients underwent needle aspiration, with subsequent incision and drainage in four of the five cases. The cultures obtained from the abscess fluids were the key to establishing the diagnosis. Only three patients (60%) received antituberculosis drugs. Wound healing lasted for a mean period of approximately seven months. Two-year follow-up was unremarkable for all of our patients.

**Conclusions:**

Technical errors continue to be significant in the development of vaccine-related complications. Bacillus Calmette-Guérin-related cold thigh abscess is an extremely rare entity.

## Introduction

Bacillus Calmette-Guérin (BCG) vaccine is considered one of the safest vaccines available [1,2]. Adverse reactions to the BCG vaccine are not common [1-6]. Factors that play a role in the development of vaccine-related complications include age and immune status of the vaccinee, route of administration and technique, and quality, strain and dose of the BCG vaccine delivered [1-5]. Technical factors are still considered the most significant in the development of vaccine-related complications [1,5]. We report five cases in which the erroneous injection of the BCG vaccine intramuscularly into the thigh lead to the formation of a cold thigh abscess.

## Case presentations

### Case 1

A six-month-old healthy Saudi girl presented with left thigh swelling that was noticed by her parents three months prior to presentation. The swelling slowly increased in size. There was no history of trauma or other illness. Our patient looked well and had normal vital signs. A physical examination revealed a soft, fluctuant, nontender left thigh lesion with no overlying skin changes. Laboratory tests revealed a white blood cell (WBC) count of 21.9 ×10^9^/L. Magnetic resonance imaging (MRI) of her left thigh revealed signs of hematoma. Needle aspiration of the lesion revealed serous, light yellowish fluids. Cultures of the aspirated fluids grew *Mycobacterium bovis*. Our patient was put on isoniazid and rifampicin for six months by the pediatric infectious disease team. At the site of the needle aspiration, our patient developed a sinus that healed within six months, leaving a small scar. The two-year follow-up was unremarkable.

### Case 2

A five-month-old healthy Saudi boy presented with left thigh swelling that was noticed by his parents three months prior to presentation. There was no history of trauma or other illness. Two weeks prior to presentation, the swelling markedly increased in size, and the overlying skin became erythematous. Our patient looked well and had normal vital signs. A physical examination revealed a fluctuant, nontender left thigh lesion with overlying skin redness and no hotness. Laboratory tests revealed a WBC count of 31.1 × 10^9^/L and an erythrocyte sedimentation rate (ESR) of 20 mm/h. MRI of his left thigh revealed an abscess. Needle aspiration of the lesion revealed purulent fluids. Incision and drainage (I&D) of the abscess were performed during the same procedure. Cultures of the aspirated fluids grew *M. bovis*. Our patient was put on isoniazid and rifampicin for six months by the pediatric infectious disease team. At the site of the I&D, our patient developed a sinus that healed within three months, leaving a small scar. The two-year follow-up was unremarkable.

### Case 3

A five-month-old healthy Saudi boy presented with left thigh swelling that was noticed by his parents three months prior to presentation. The swelling slowly increased in size. There was no history of trauma or other illness. Our patient looked well and had normal vital signs. A physical examination revealed a soft, fluctuant, nontender left thigh lesion with no overlying skin changes. Laboratory tests revealed a WBC count of 16.8 × 10^9^/L and an ESR of 13 mm/h. An ultrasound of his left thigh indicated an abscess. Needle aspiration of the abscess revealed purulent fluids. I&D of the abscess were performed during the same procedure. Cultures of the aspirated fluids grew *M. bovis*. Our patient was put on isoniazid and rifampicin for six months by the pediatric infectious disease team. However, the medications were stopped after three months because our patient developed an impaired level of consciousness. At the site of the I&D, our patient developed a sinus that became infected with *Pseudomonas *and required another I&D procedure. Wound healing lasted for ten months, leaving a small scar. The two-year follow-up was unremarkable.

### Case 4

A nine-month-old healthy Saudi boy presented with left thigh swelling that was noticed by his parents three months prior to presentation. The swelling slowly increased in size. Ten days prior to presentation, the overlying skin became erythematous, and the lesion increased in size. There was no history of trauma or other illness. Our patient looked well and had normal vital signs. A physical examination revealed a fluctuant, nontender left thigh lesion with overlying skin erythema. Laboratory tests revealed an ESR of 55 mm/h. Needle aspiration of the lesion revealed purulent fluids. I&D of the abscess were performed during the same procedure. Cultures of the aspirated fluids grew *M. bovis*. Our patient did not receive antituberculosis drugs. At the site of the I&D, our patient developed a sinus that healed within eight months, leaving a small scar. The two-year follow-up was unremarkable.

### Case 5

An eight-month-old healthy Saudi boy presented with left thigh swelling that was noticed by his parents four months prior to presentation. The swelling slowly increased in size. There was no history of trauma or other illness. Our patient looked well and had normal vital signs. A physical examination revealed a fluctuant, nontender left thigh lesion with no skin changes. Laboratory tests revealed a WBC count of 23.8 × 10^9^/L. Needle aspiration of the lesion revealed purulent fluids. I&D of the abscess were performed during the same procedure. Cultures of the aspirated fluids grew *M. bovis*. Our patient did not receive antituberculosis drugs. At the site of the I&D, our patient developed a sinus that healed within eight months, leaving a small scar. The two-year follow-up was unremarkable.

## Discussion

Thigh swelling is a symptom of various benign and malignant conditions [7-9]. Hematoma as a result of trauma, iatrogenic injection or coagulopathy is one of the causative disorders. Infections like cellulitis, necrotizing fasciitis, septic arthritis, and osteomyelitis are other causes of thigh swelling in infants. Benign and malignant tumors can also present as thigh swelling. Differential diagnosis is not always easy, particularly if the causative disorder has not previously been reported.

In the first case, there was no history of witnessed trauma. The rest of the medical and family history was also unremarkable, making the diagnosis of hematoma less likely. There were no general or local sign of infection. Tumor was the most likely diagnosis on physical examination. MRI of her left thigh did not provide a clear answer but revealed signs of hematoma and indicated that needle aspiration be used for a final diagnosis. When the cultures grew *M. bovis*, it became obvious that the abscess was a complication of BCG vaccination. However, the pathogenesis was not clear. The absence of skin scarring in the left upper arm above the deltoid muscle in our patients raised the possibility of inadvertent injection of the BCG vaccine intramuscularly into the left thigh instead of the intradermal administration into the left upper arm. Our patient was clinically well. However, the possibility of subclinical disseminated disease in this case and in the two subsequent cases was the justification for prescribing antituberculosis medications given by the pediatric infectious disease team.

In the rest of the cases, the possibility of a BCG-related cold thigh abscess was considered. The cultures of the aspirated fluids were the key to establishing the final diagnosis.

In the Kingdom of Saudi Arabia, as per World Health Organization recommendations, the BCG vaccine is given to all healthy neonates [5,10]. The technique involves the intradermal injection of 0.05 mL of the vaccine into the left upper arm. In our hospital, BCG vaccine is given to all healthy newborns three to four hours after birth intradermally in the left upper arm. The vaccine used is manufactured by Statens Serume Institut, Denmark. At the same time, 0.5 mL of hepatitis B vaccine is injected intramuscularly in one thigh and vitamin K into the other thigh. All these injections used to be given by one certified nurse in the same room and at the same time.

A review of the medical records revealed that all of these infants were vaccinated by the same nurse, who is certified and has experience. Investigations did not reveal how the error occurred; however, the only reasonable explanation is that the BCG vaccine was confused with the hepatitis B vaccine or vitamin K, and inadvertently injected intramuscularly into the left thigh instead of an intradermal administration into the left upper arm. The recommendation of the infection control and patient safety committees was to assign one nurse to give the BCG vaccine in an assigned BCG vaccination room and another nurse to administer the hepatitis B vaccine and the vitamin K in a different room and at an another time. Since that policy was implemented approximately three years ago, we have not had any similar cases.

The BCG vaccine is administered to 100 million children each year and is considered to be one of the safest vaccines available [1,2]. Adverse reactions arising from the use of BCG vaccine in healthy infants are relatively uncommon [1-6]. Local and regional adverse reactions to BCG vaccinations include local swelling, abscess formation, ulceration, keloid formation and regional lymphadenitis. Such reactions occur at a rate of four-30 per 1000 vaccinated infants and comprise the majority (84%) of adverse reactions reported [1,4]. Local and regional adverse reactions are generally self-limiting and require no treatment [2,3]. Systemic adverse reactions to the BCG vaccine are rare and include osteomyelitis and disseminated BCG infection [1,4]. Disseminated BCG infection has been estimated to occur at a rate of 0.19-1.56 per million vaccinees and is associated with a high case fatality ratio of 80-83%. Disseminated BCG infection has almost exclusively occurred in individuals with severely compromised cellular immunity. However, it also has also been reported in healthy immunocompetent infants [5]. Vaccine-induced extensive ulcerating vasculitis is also an extremely rare systemic adverse reaction to the BCG vaccine [6].

The considerable variation in the number of adverse reaction reports from different countries is thought to be due to a number of factors, including the level of case finding and the diagnostic criteria employed, the route of administration and technique, the age and immune status of the vaccinees and the quality, strain and dose of the BCG vaccine delivered [1-5]. Many reports have stressed the importance of technical factors [1,5]. One of these technical errors is subcutaneous administration of the BCG vaccine, which can result in localized abscess formation. Other technical issues involve incorrect reconstitution of the vaccine, incorrect selection of syringes and the use of the same syringe to vaccinate several children, leading to errors in dose.

To the best of our knowledge, BCG-related cold thigh abscess has not previously been reported in the English literature. In our case series, the only reasonable explanation for the phenomenon observed is the confusion of the BCG vaccine for the hepatitis B vaccine and/or vitamin K, and the inadvertent injection of the BCG vaccine intramuscularly into the thigh by the vaccinating nurse. The number of newborns that received the BCG vaccine intramuscularly into the thigh is unknown. It is also unknown how many of them developed local reactions and how many were treated at other centers. It is also unclear whether other factors, such as age of the vaccinees or the quality of the vaccine strain, played a role in abscess formation in our patients. Nevertheless, we think that the technical error of confusing the vaccines and administering the BCG vaccine intramuscularly played the main role in our reported cases.

## Conclusions

Correct vaccination techniques remain the most important factor in the prevention of vaccine-related complications. Different vaccines should not be given simultaneously by the same nurse in the same room. BCG-related cold thigh abscess as a result of erroneous administration of the BCG vaccine intramuscularly into the thigh is an extremely rare occurrence.

## Consent

Written informed consents were obtained from the parents of our patients for publication of this case series. Copies of the written consents are available for review by the Editor-in-Chief of this journal.

## Competing interests

The authors declare that they have no competing interests.

## Authors' contributions

OO and JA analyzed and interpreted the patient data. SJ, SC, EB, MS, and MZ were major contributors in writing the manuscript. All authors read and approved the final manuscript.
